# Editorial: Nanostructured steady-state systems for nutrient and bioactive compounds delivery

**DOI:** 10.3389/fnut.2024.1421572

**Published:** 2024-05-21

**Authors:** Mingqian Tan, A. M. Abd El-Aty

**Affiliations:** ^1^State Key Laboratory of Marine Food Processing and Safety Control, Dalian Polytechnic University, Dalian, China; ^2^Department of Pharmacology, Faculty of Veterinary Medicine, Cairo University, Giza, Egypt

**Keywords:** nanostructured steady-state systems, delivery, bioavailability, bioactive compounds, nutrients

In an era where health and wellness are paramount concerns, the exploration of innovative ways to deliver essential nutrients and bioactive compounds holds great promise. Among these approaches, nanoencapsulation technology stands out as a particularly effective means of enhancing the bioaccessibility and stability of vital nutritional components. Within this field, the emergence of nanostructured, steady-state systems represents a compelling avenue for exploration and development.

Steady-state nanostructured systems based on food-grade materials offer a versatile approach for the delivery of nutrients and bioactive compounds. By leveraging nanotechnology, these systems exhibit characteristics such as small size, high permeability and robust stability, which together help to overcome barriers to effective delivery. The encapsulation of essential nutrients, ranging from peptides and minerals to vitamins and polyunsaturated fatty acids, within nanostructured carriers holds promise for preserving their integrity against physical, chemical, and enzymatic degradation (1).

In addition, nanostructured, steady-state systems offer additional benefits beyond simple preservation. They have the potential to mitigate undesirable tastes and odors, enhance bioavailability, and facilitate controlled release dynamics, thereby optimizing the delivery of bioactive compounds (2). Central to this paradigm is the use of food-derived proteins and polysaccharides in the construction of nanoparticulate encapsulates, underscoring the importance of biocompatible materials in the food industry's quest for functional delivery solutions (3).

The current research landscape is rich with investigations of various nanostructured systems, including nanoemulsions, micelles, lipid-based nanoparticles, and biopolymer-based carriers, among others. These efforts span both academic and engineering domains and seek to elucidate the intricacies of preparation techniques and the nuanced effects of preparation conditions on system performance.

Recent contributions to this field have shed light on promising avenues for nutrient delivery. For example, the use of hydrogels as carriers for bioactive food ingredients is compelling. These three-dimensional polymeric networks offer versatility and resilience, providing a platform for embedding a wide range of nutraceuticals while overcoming the challenges of the gastrointestinal tract, as reviewed by Li et al.. In addition, the encapsulation of bioactive compounds extracted from date palm seeds in liposomes has the potential to enhance bioavailability and functionality in functional foods, exemplifying the tangible impact of nanoencapsulation in real-world applications, as reported by Hashim et al..

Similarly, the exploration of nanocarrier systems for the delivery of bioactive peptides underscores the transformative potential of nanotechnology for enhancing bioavailability and biostability, as shown by Zhang et al.. By utilizing a spectrum of delivery systems ranging from liposomes to hydrogels, researchers are paving the way for maximizing the utilization of bioactive peptides in various fields. Furthermore, Shakeri et al. encapsulated vitamin D3 (VD3) and omega-3 (ω3) in beeswax solid lipid nanoparticles (BW. SLNs) for improved stability in functional foods. They achieved high encapsulation efficiency at specific concentrations, with favorable physicochemical properties. Release studies revealed differential kinetics, and the encapsulated compounds remained stable under various conditions and during storage. Coloaded VD3 and ω3 in BW. SLNs have potential for use in food fortification and functional food production.

As we delve deeper into the realm of nanostructured, steady-state systems, it is imperative to take a multidisciplinary approach. Collaboration across scientific disciplines, from food science and technology to materials engineering and pharmacology, will be critical to unlocking the full potential of these transformative technologies.

In conclusion, nanostructured steady-state systems offer a paradigm shift in the delivery of essential nutrients and bioactive compounds. Through careful research and innovative design, these systems promise to revolutionize the functional food and nutraceutical landscape, ultimately contributing to improved health and wellbeing on a global scale.

## Author contributions

MT: Writing – original draft, Writing – review & editing. AA: Writing – original draft, Writing – review & editing.
